# Detection of bitterness and astringency of green tea with different taste by electronic nose and tongue

**DOI:** 10.1371/journal.pone.0206517

**Published:** 2018-12-31

**Authors:** Guangyu Zou, Yanzhong Xiao, Miaosen Wang, Hongmei Zhang

**Affiliations:** School of Mechanical and Electrical Engineering, Henan Agricultural University, Zhengzhou, Henan, China; Duke University School of Medicine, UNITED STATES

## Abstract

An electronic nose was used to evaluate the bitterness and astringency of green tea, and the possible application of the sensor was assessed for the evaluation of different tasting green tea samples. Three different grades of green tea were measured with the electronic nose and electronic tongue. The sensor array of the E-nose was optimized by correlation analysis. The relationship between the signal of the optimized sensor array and the bitterness and astringency of green tea was developed using multiple linear regression (MLR), partial least squares regression (PLSR), and back propagation neural network (BPNN). BPNN is a multilayer feedforward neural network trained by an error propagation algorithm. The results showed that the BPNN model possessed good ability to predict the bitterness and astringency of green tea, with high correlation coefficients (R = 0.98 for bitterness and R = 0.96 for astringency) and relatively lower root mean square errors (RMSE) (0.25 for bitterness and 0.32 for astringency) for the calibration set. The R value is 0.92 and 0.87, and the RMSE is 0.34 and 0.55, for bitterness and astringency, respectively, of the prediction set. These results indicate that the electronic nose could be used as a feasible and reliable method to evaluate the taste of green tea. These results can provide a theoretical reference for rapid detection of the bitter and astringent taste of green tea using volatile odor information.

## Introduction

Xinyang Maojian tea, which is one of the ten famous teas in China, occupies a very important position in Chinese tea culture. It is famous for its unique fine, round, light, hairy leaves that are highly fragrant with a rich, green color [1,2]. The flavor components of Xinyang Maojian tea are mainly composed of polyphenols, caffeine, amino acids, and dozens of organic compounds. These substances are also the main factors that contribute to the bitter and astringent taste of tea. Tea polyphenols are the general term for polyhydroxyphenol derivatives, and the main flavors of these compounds are bitter and astringent.

Catechins account for approximately 70%-80% of total tea polyphenols. The four components in tea with the highest catechin content are epicatechin (EC), epicatechin gallate (ECg), epigallocatechin (EGC), and epigallocatechin gallate (EGCg) [3]. According to recent studies, sensory evaluations indicated that the taste of catechin is bitter and astringent, and the threshold of eight types of catechin bitterness and astringency has been determined [4]. Some studies have reported sensory characteristics of catechins in tea, and the results suggest that ECg might be recognised by taste cells [5]. Flavonoids also have a bitter taste or an enhanced bitterness [6]. Another important factor affecting the bitter taste of tea is caffeine. Caffeine is the main alkaloid in tea, which generally accounts for 2%-5% of the dry weight of tea. Some studies have shown that the main flavour of caffeine is bitterness[7,8]. In addition, a variety of free amino acids in tea also influence its taste. Previous studies that have evaluated the flavor characteristics of some amino acids in tea have determined that different amino acids have different flavors, such as bitter, sweet, fresh, and astringent [9]. The main source of bitterness in tea is caffeine, and the main source of astringency in tea is catechins. Different chemical components make different kinds of bitterness and astringent, which is one of the reasons for distinguishing tea from other drinks.

The bitterness and astringency of tea are mainly formed by the combination of tea polyphenols, caffeine, and some amino acids [4,10]. Thus far, the methods to evaluate the taste of tea mainly include sensory evaluation [11], chemical composition analysis [12], taste dilution analysis [6], and electronic tongue analysis [13]. These methods have the disadvantages of human factor error, complex operation, and long detection time. The electronic nose is used as a biomimetic olfactory instrument that can be used to quickly collect and evaluate the odor information of samples. It has been applied to many fields of research for food quality detection, such as wheat [14], fruits [15], meat [16], wine [17], and tea [18,19]. All these previous studies have demonstrated the feasibility of using an electronic nose to appraise food quality. Therefore, the current study used electronic nose technology to model the bitterness and astringency of tea infusions. The tea flavor information was collected to evaluate the bitter and astringent taste of tea. The current study presents a new method that can be used to judge the taste of tea infusions.

## Materials and methods

### Materials

The Xinyang Maojian tea samples were picked before the Qingming Festival, in Xinyang district, Henan province in April, 2017. Three different grades of tea were used in the experiment. All grades of tea were stored in tin foil bags. Before the experiment, the tea was refrigerated at 4°C.

### Electronic nose

A portable electronic nose (PEN3) from Win Muster Airsense (WMA) Analytics Inc. was used. The main components of the electronic nose are a sensor array, sampling and cleaning channel, data acquisition system, and computer. The sensor array is composed of ten metal oxide semiconductor (MOS) sensors. These ten MOS sensors operate at 350–500°C. The response of each sensor used in this study is given in Table 1. The response value measured by the sensor is the resistance ratio, which corresponds to a type of odor of the tea. The output of the sensor is G/G0, where G is the conductivity of the volatile gas, and G0 is the conductivity of the gas filtered by standard activated carbon. The electronic nose uses WinMuster software to collect, measure, and analyze the data, and stores the collected data on a computer.

**Table 1:** Response features of the sensor array.

Sensor name	Response of sensor
W1C	benzene aromatic ingredient
W5S	highly sensitive to oxynitride
W3C	sensitive to ammonia aromatic ingredient
W6S	selective about hydride
W5C	sensitive to methane, propane and aliphatic non-polar molecules
W1S	sensitive to methyl-like
W1W	sensitive to inorganic sulfide
W2S	sensitive to alcohols and aldehydes
W2W	sensitive to organic sulfide aromatic ingredient
W3S	sensitive to methane and aliphatics

### Electronic tongue

This study used Taste-Sensing System SA402B, which was produced by Intelligent Sensor Technology Co. Ltd. in Japan. The electronic tongue is mainly composed of three parts: the detection instrument, the sensor array, and the operating computer. The sensor array is composed of taste sensors and reference electrodes. Taste sensors include bitterness and astringency sensors, which consist of a sensor probe body, artificial biomolecular ester film, Ag/AgCl electrode, and pin-inserted electrode terminal. The artificial ester film used for ion exchange is only a few hundred micrometers thick. An Ag/AgCl reference electrode was installed on each sensor head to measure the change in the membrane potential after the sensors were immersed in the sample solution, and mV values were consequently obtained. The sample value was determined relative to a preliminarily measured standard solution consisting of potassium chloride and tartaric acid. After sample measurement and two short cleaning steps, the taste value was measured by immersing the sensors into the standard solution. The adsorption of the substance to the membrane was measured, and the obtained value is called CPA value (“change of membrane potential caused by adsorption”). The electronic tongue’s own software can automatically convert the measured potential into a taste value.

### Experiment with the electronic nose and tongue

Before the electronic nose test, the tea sample was placed in a 250-mL beaker and was sealed for 45 min with a double layer of preservative film. The electronic nose was used to detect the aroma of tea leaves by headspaces solid phase microextraction. Sampling occurred every second, continuously for 60 s. The data were obtained with an odor sensor response that returned to baseline throughout the experiment with a cleaning time of 50 seconds.

The electronic tongue was used to detect the bitterness and astringency of tea. The tea brewing method that was used is as follows: 5.00 g of tea leaves were added to a teapot. Then, 250 mL of boiling deionized water was poured into the teapot, and the mixture was allowed to stand for 5 min at ambient temperature (25°C±2°C). After the brewing, the tea leaves were filtered out, and the proportion of tea and water was 1:50 (g/mL); the liquid was cooled to ambient temperature before the experiment. To collect the data, the taste sensor response returned to baseline throughout the experiment with a cleaning time of 5.5 minutes. The sample taste test time was 30 s, and sample aftertaste time was 30 s.

### Data and statistical analyses

The electronic tongue was used for taste evaluation. A correlation analysis was used to optimize the electronic nose sensor array. The sensor array of the electronic nose was optimized by eliminating the sensors with poor correlation. The response value of the electronic nose sensor array was used as the input, and the bitterness and astringency value obtained by the electronic tongue was used as the output for mathematical modeling. Three different models were established using multiple linear regression (MLR), partial least squares regression (PLSR), and back propagation neural network (BPNN). Comparing the regression coefficient and the root mean square error of the models, the most optimal prediction model was obtained. The modeling was performed using MATLAB 2012a software.

## Experimental results and analysis

### Data preprocessing

**Fig 1:**
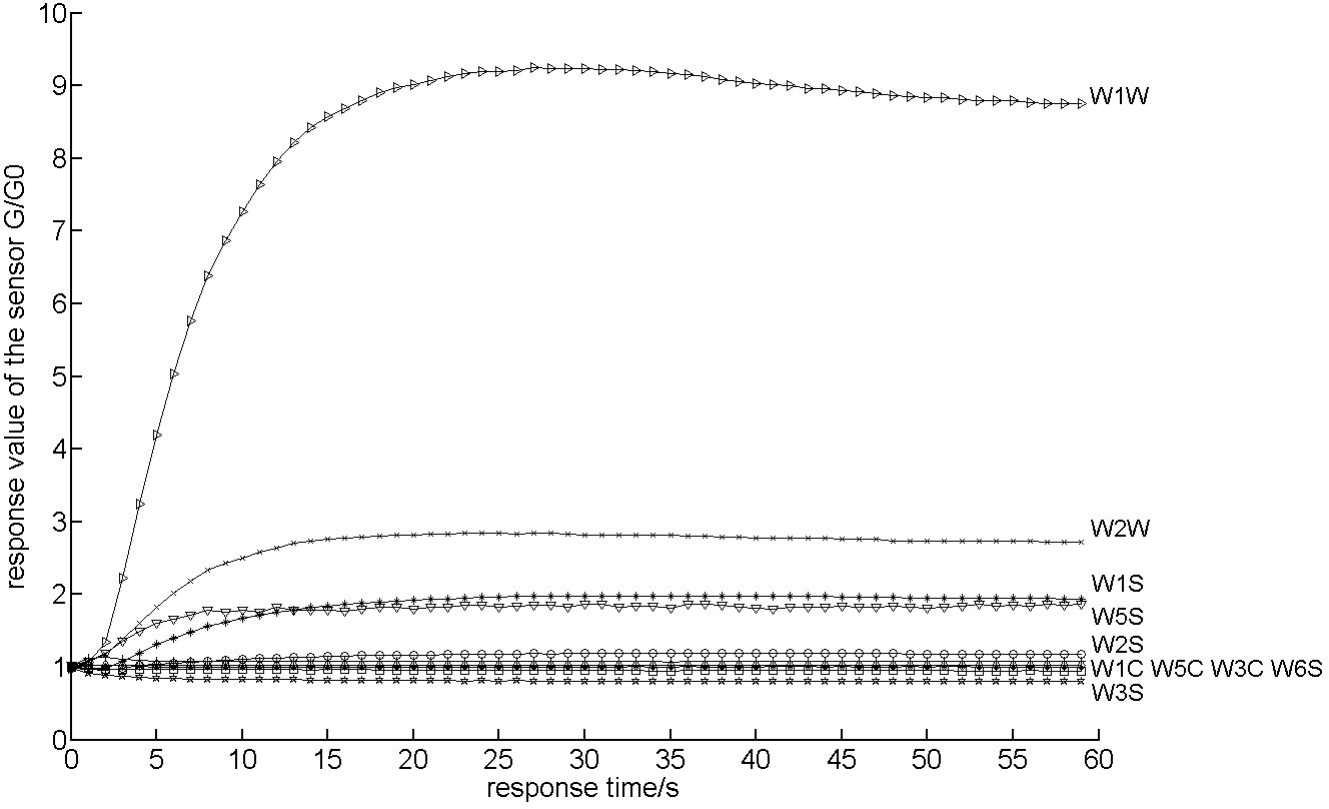
Response of sensor array to tea odor.

Fifteen repeated tests were conducted for each grade tea sample. Thus, as shown in Fig 1, an original data matrix of size 10 × 60 was obtained in each test of the electronic nose. The total matrix size of all grade tea samples is 600 × 45. Because the original matrix information was too large for machine learning, it was necessary to reduce the data dimension by extracting data features. Fig 1 shows that the response of each sensor began to stabilize at 30 s. Therefore, the average value was extracted as the characteristic value, when the sensor response was stable. The new matrix size after feature extraction was 10 × 45.

### Chemical composition and taste analysis of green tea

Polyphenols and caffeine are two important factors that affect the aroma and taste of tea. As shown in Table 2, the polyphenol content was significantly changed in green tea of different grades. The polyphenol content was positively correlated with the quality of tea. The caffeine content in top grade tea was the lowest, and the caffeine content in first and second grade tea was similar. The caffeine content also was positively correlated with tea quality. Tea polyphenols and caffeine affect the aroma and taste of tea, and they greatly contribute to the formation of tea aroma and flavor diversity. Yashin reported that polyphenols affect the volatility of flavor compounds[20]. Some studies have shown that chemical reactions between caffeine and flavor compounds can form a molecular complex[21]. Zhou has found that the aroma and taste of tea can be altered by adding exogenous amino acids[22]. In addition, the bitterness value of the tea measured by the electronic tongue was proportional to the astringent value. The bitterness of second grade tea was the greatest, and the bitterness of the top grade tea was moderate.

**Table 2:** Chemical composition and taste analysis of Xinyang Maojian tea.

Sample	Mass fraction of polyphenol /%	Mass fraction of caffeine /%	Bitterness value	Astringency value
Tea in top grade	21.02%	3.78%	5.63	26.05
Tea in first grade	19.58%	3.72%	4.50	25.20
Tea in second grade	18.81%	3.52%	6.24	27.50

### Correlation analysis and optimization of the sensor array

Electronic nose measures flavor components affected by bitter and astringent substances of tea, while electronic tongue measures the bitter and astringent values of tea soup. The correlation between the response value of the electronic nose and tongue was analyzed, and the results are shown in Table 3. The significance value was less than 0.05, indicating that the response of the sensor was related to the bitterness and astringency [10]. Table 3 shows that significant values between some sensors (W1C, W3C, W2S) and the bitterness (or astringency) were greater than 0.05. These sensors were not conducive to the prediction of the bitterness and astringency of the tea. Therefore, sensors W1C, W3C, W2S were eliminated, and a new sensor array was obtained to predict the bitterness and astringency of the tea infusion.

**Table 3:** Significance analysis between electronic nose and tongue.

	W1C	W5S	W3C	W6S	W5C	W1S	W1W	W2S	W2W	W3S
bitterness	0.706	0.018	0.396	0.006	0.003	0.462	0.000	0.300	0.000	0.021
astringency	0.732	0.020	0.609	0.093	0.149	0.045	0.000	0.082	0.000	0.359

### Establishing bitter and astringent models

MLR is a statistical method to study the linear effect of multiple independent variables on dependent variables. The relationship between the independent variables and the dependent variables must be linear [23]. MLR analysis is convenient and fast, and all the independent variables can be taken into account. The method of establishing the MLR model is as follows: the response values of the seven sensors in the electronic nose were used as independent variables. The bitterness and astringency values of the tea detected by the electronic tongue were used as dependent variables. Ten of the fifteen test results of each grade of tea samples were randomly selected as the calibration set and the remaining five values were used as the prediction set. A total of 30 data entries were used to train the MLR model, and the remaining 15 data entries were used to test the prediction effect of the model.

**Fig 2:**
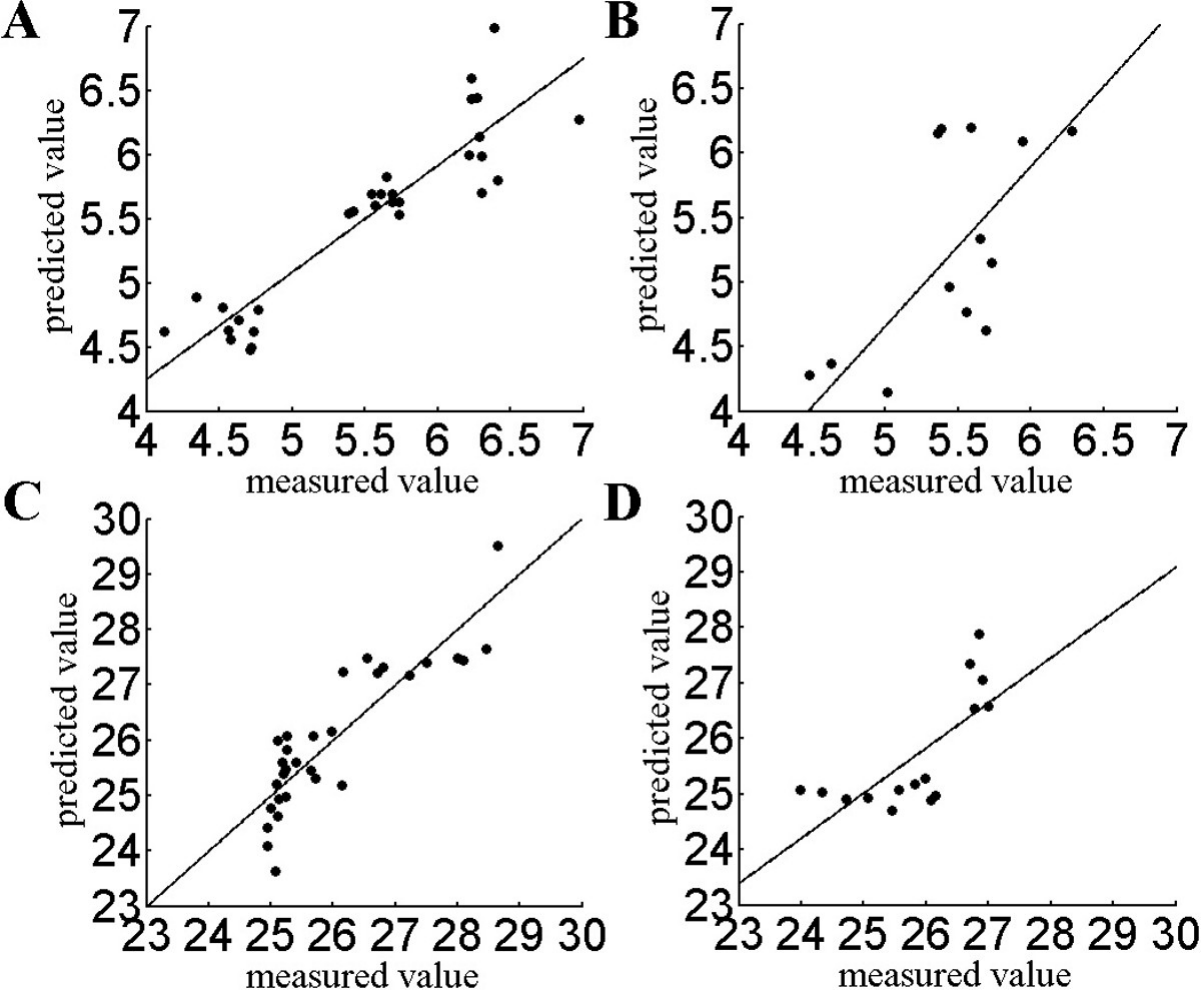
Establish the MLR model for the bitter and astringent taste of tea. a. Calibration set for the bitter taste of tea b. Prediction set for the bitter taste of tea c. Calibration set for the astringent taste of tea d. Prediction set for the astringent taste of tea.

The MLR model is shown in Eqs 1 and 2. Fig 2 shows the comparison between the predicted results of the MLR model and the bitterness (or astringency) of tea as measured by the electronic tongue. The MLR model performed effectively in predicting the bitterness and astringency of the tea based on the electronic nose responses obtained from Xinyang Maojian tea. The root mean square error and fitting correlation coefficients of the bitterness (RMSE = 0.309, R = 0.833) calibration set were slightly better than those of the astringent (RMSE = 0.618, R = 0.77) calibration set. The root mean square error and fitting correlation coefficients of the bitterness (RMSE = 0.643, R = 0.558) prediction set were also slightly better than those of the astringent (RMSE = 0.73, R = 0.542) prediction set. The MLR calculations are as follows:

$${\text{MLR}}_{\text{bit}}=-1.91+0.329\times {\text{X}}_{1}+6.97\times {\text{X}}_{2}+6.013\times {\text{X}}_{3}+1.014\times {\text{X}}_{4}-0.007\times {\text{X}}_{5}+0.207\times {\text{X}}_{6}-10.314\times {\text{X}}_{7}$$ (1)

$${\text{MLR}}_{\text{ast}}=1.785-1.823\times {\text{X}}_{1}+33.683\times {\text{X}}_{2}-0.596\times {\text{X}}_{3}-1.247\times {\text{X}}_{4}+0.348\times {\text{X}}_{5}+0.11\times {\text{X}}_{6}-7.092\times {\text{X}}_{7}$$ (2)

where MLR_bit_ is the predictive value for the MLR model for bitterness, MLR_ast_ is the predictive value for the MLR model for astringency, and X1-X7 correspond to the response values of sensor W5S, W6S, W5C, W1S, W1W, W2W, and W3S, respectively.

PLS is a fixed linear regression technique that reduces the size of the variables by extracting linear combinations from the originals [24]. PLSR is based on multiple linear regression analysis, combined with principal component analysis and canonical correlation analysis, and can provide a more reasonable regression model. The PLSR method is similar to principal component regression analysis, except that both the target vector and the measurements are used to determine a lower-dimensional subspace from which the predictions will be made [25]. With the above method, 30 data entries were taken as calibration sets, 15 data entries were used as prediction sets, and a PLSR model was established.

**Fig 3:**
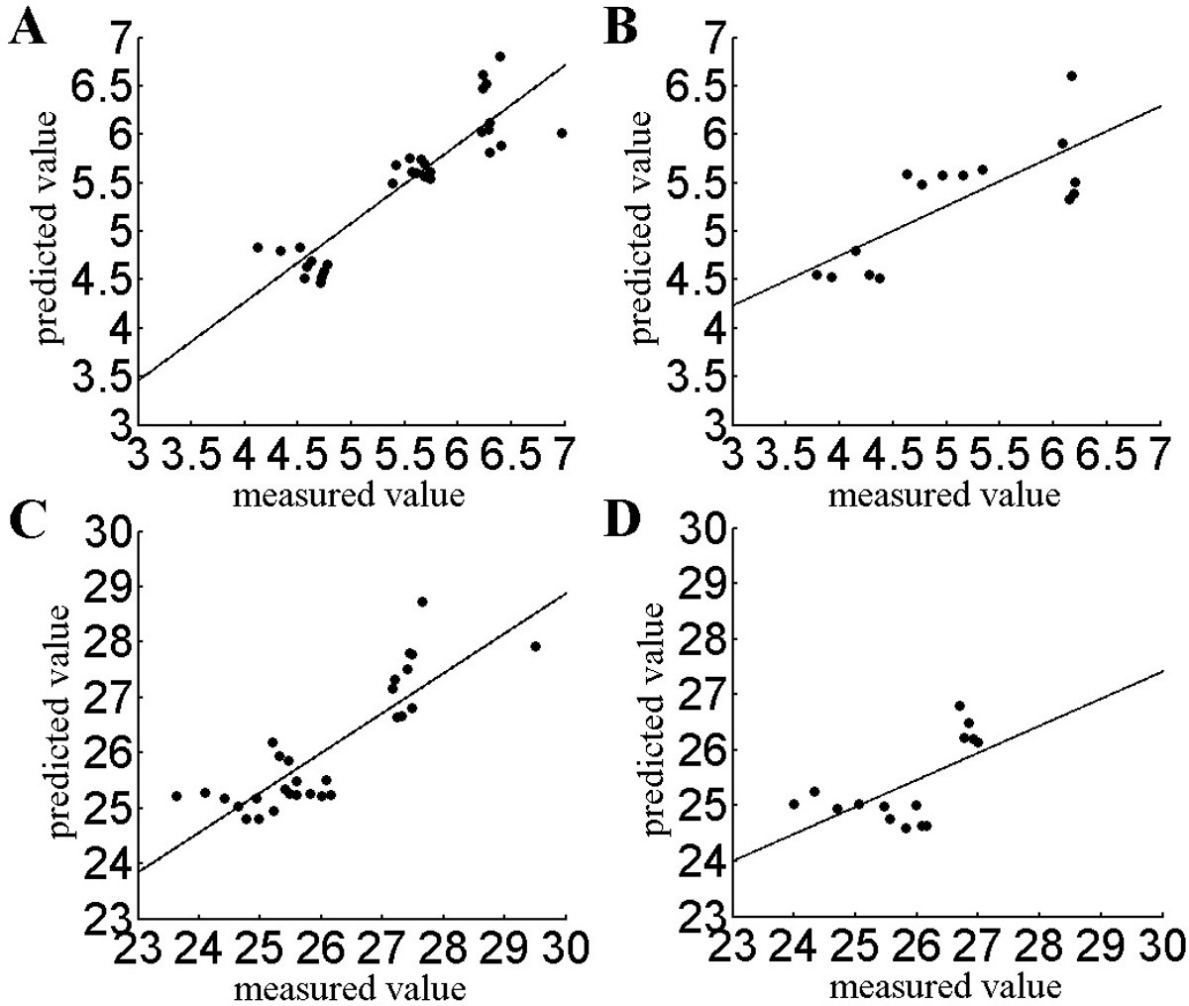
Establish the PLSR model for the bitter and astringent taste of tea. a. Calibration set for the bitter taste of tea b. Prediction set for the bitter taste of tea c. Calibration set for the astringent taste of tea d. Prediction set for the astringent taste of tea.

The PLSR model is shown in Eqs 3 and 4. Fig 3 shows the comparison between the prediction results of the PLSR model and the bitterness (or astringency) of tea as measured by the electronic tongue. The PLSR model has a general effect on the prediction of bitter taste, although the prediction effect for astringency is not as robust. The root mean square error and fitting correlation coefficients of the bitterness (RMSE = 0.326, R = 0.815) calibration set were slightly better than those of the astringent (RMSE = 0.686, R = 0.718) calibration set. The root mean square error and fitting correlation coefficients of the bitterness (RMSE = 0.61, R = 0.58) prediction set were also slightly better than those of the astringent (RMSE = 0.868, R = 0.386) prediction set. The PLSR calculations are as follows:

$${\text{PLSR}}_{\text{bit}}=-7.125+0.533\times {\text{X}}_{1}-10.732\times {\text{X}}_{2}+21.975\times {\text{X}}_{3}+0.054\times {\text{X}}_{4}+0.031\times {\text{X}}_{5}+0.172\times {\text{X}}_{6}-1.983\times {\text{X}}_{7}$$ (3)

$${\text{PLSR}}_{\text{ast}}=43.046-1.315\times {\text{X}}_{1}-17.301\times {\text{X}}_{2}-0.528\times {\text{X}}_{3}+0.512\times {\text{X}}_{4}+0.133\times {\text{X}}_{5}+0.378\times {\text{X}}_{6}-1.894\times {\text{X}}_{7}$$ (4)

where PLSR_bit_ is the predictive value of the PLSR model for bitterness, PLSR_ast_ is the predictive value of the PLSR model for astringency, and X1-X7 correspond to the response values of the sensor W5S, W6S, W5C, W1S, W1W, W2W, W3S respectively.

**Fig 4:**
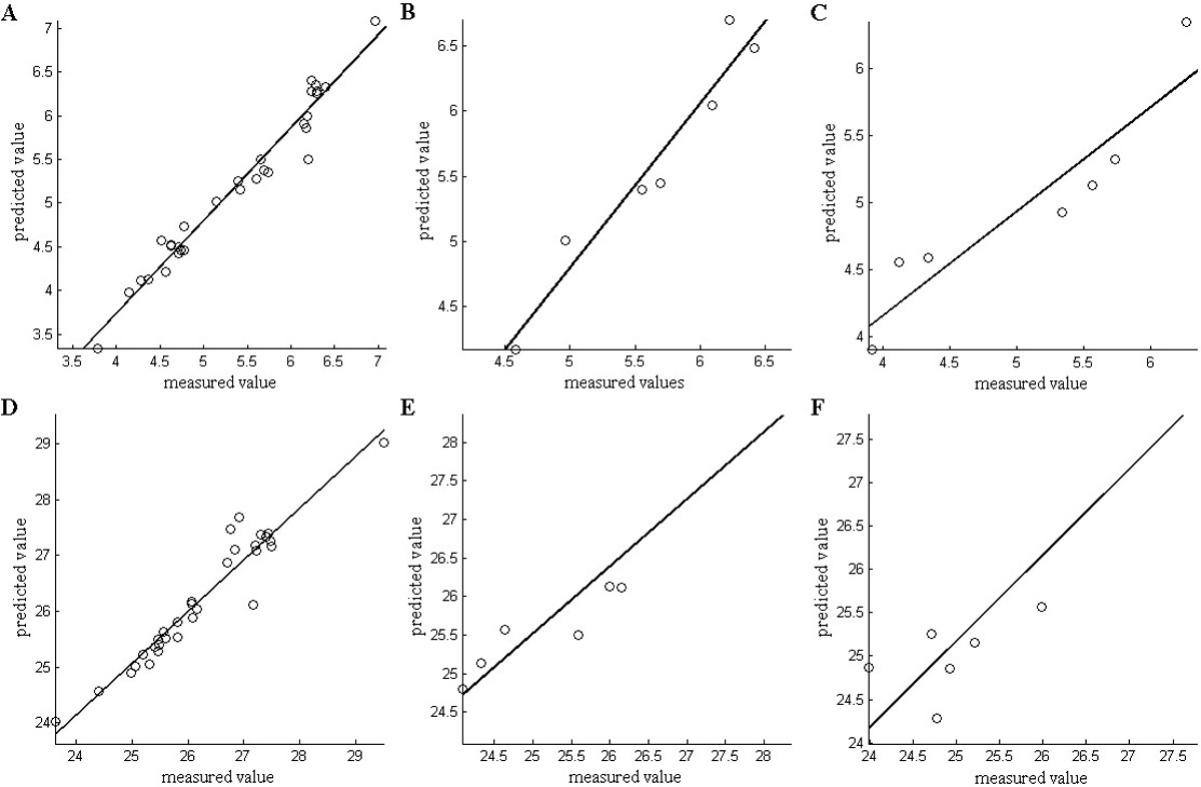
Establish the BPNN model for the bitter and astringent taste of tea. a. Calibration set for the bitter taste of tea b. Validation set for the bitter taste of tea c. Prediction set for the bitter taste of tea d. Calibration set for the astringent taste of tea e. Validation set for the bitter taste of tea f. Prediction set for the astringent taste of tea.

The BP neural network has a strong ability to construct input and output maps, and mathematical expressions are not required to describe the mapping relationship between the input and output. The BPNN model uses the sensor response value of tea as the input value [26]. A BPNN has the advantages of high reliability of simulation results. The bitterness and astringency values of tea are regarded as target values. Next, 70% of the input value was considered as the calibration set, 15% as the validation set, and the remaining 15% as the prediction set. The number of hidden layers of neurons was set to 10 levels for training. Fig 4 shows the comparison between the prediction results of the BPNN model and the bitterness (or astringency) of tea measured by the electronic tongue. The BPNN model performed very effectively in predicting the bitterness and astringency of tea based on the electronic nose responses obtained from Xinyang Maojian tea. The root mean square error and fitting correlation coefficients of the bitterness (RMSE = 0.255, R = 0.981) calibration set were slightly better than those of the astringent (RMSE = 0.318, R = 0.961) calibration set. The root mean square error and fitting correlation coefficients of the bitterness (RMSE = 0.34, R = 0.923) prediction set were also slightly better than those of the astringent (RMSE = 0.547, R = 0.866) prediction set.

The regression coefficient and root mean square error of the calibration set and prediction set were used as evaluation indexes, and the results are shown in Tables 4 and 5. The three types of models have a satisfactory prediction effect on the calibration set of bitter taste. However, the regression coefficient of the prediction set indicates that the MLR and PLSR model have poor practical application, and the adaptability of the new data is weak. This may be due to less sample data from the calibration set. The BPNN model has good learning ability. In the recognition of the bitter taste of tea, the calibration set and prediction set have the most optimal effect. In the evaluation of the astringency of tea, the recognition effect of the MLR and PLSR model is poor, although the BPNN model still retains excellent recognition ability. In summary, the BPNN model has a larger regression coefficient and a smaller root mean square error in the recognition of the bitterness and astringency of tea. In this study, the BPNN was the most optimal model for the prediction of bitter taste and astringency.

**Table 4:** Comparison of the prediction results of three models for bitterness.

Model	Regression coefficient of calibration set	Regression coefficient of prediction set	Root mean square error of calibration set	Root mean square error of prediction set
MLR	0.833	0.558	0.309	0.643
PLSR	0.815	0.58	0.326	0.61
BPNN	0.981	0.923	0.255	0.34

**Table 5:** Comparison of the prediction results of three models for astringency.

Model	Regression coefficient of calibration set	Regression coefficient of prediction set	Root mean square error of calibration set	Root mean square error of prediction set
MLR	0.77	0.542	0.618	0.73
PLSR	0.718	0.386	0.686	0.868
BPNN	0.961	0.866	0.318	0.547

## Conclusions

A correlation analysis between an electronic nose and electronic tongue can effectively optimize the electronic nose sensor array, reduce the difficulty of data modeling, and improve the efficiency of machine recognition. Based on the analysis of the bitterness and astringency of tea by the optimized electronic nose sensor array, the BPNN model is more accurate than the MLR and PLSR model. The regression coefficient of the BPNN model calibration set was greater than 0.95, the regression coefficient of prediction set was greater than 0.85, and the overall root mean square error was also small. This indicates that the BPNN model has a great effect on the recognition of bitterness and astringency of tea. BP neural network has nonlinear mapping ability, self learning ability, adaptive ability, generalization ability and fault tolerance. Neural network structure can be adjusted through iterative learning. It is feasible to use an electronic nose to recognize the bitterness and astringency of a tea infusion. This study provides a new method for judging the bitterness and astringency of tea infusions.
